# Surgery

**Published:** 1838-01

**Authors:** 


### SURGERY.

*Case of partial Paralysis of the Face from Injury of the Head, with Observations.* By ALEXANDER SHAW, Esq., Assistant Surgeon to the Middlesex Hospital.

THIS is a very interesting and valuable case, and the remarks appended to it constitute an important addition to the pathology of partial paralysis of the face, as well as to the physiology of the parts concerned. A man, aged twenty, was admitted into the Middlesex Hospital on the 3d September, having fallen from a railroad-carriage in a state of intoxication.

"When brought to the hospital he lay comatose, breathing heavily, occasionally vomiting, and when roused, he struggled and became noisy. There was an open contused wound of the scalp, near the tuberosity of the occipital bone, a little to its left. Blood flowed freely from the right ear." . . . "6th. The muscles of the right side of the face (except those that move the jaw) are found to be paralyzed. On a close examination, these are the only parts similarly affected. The sensibility of the side of the face is not impaired." . . . "7th. The symptoms are nearly the same. Although the distortion of the features is more remarkable than it was yesterday, the right eye is closed as in natural sleep; and even after the eyelid is raised, it descends again, so as to cover the eye." . . . "13th. The features are now so near being equally balanced, that the distortion of the face can only be perceived with distinctness when he is roused, and uses the right side of his face.

"16th. All his symptoms are improved, but the amendment in his face has not continued." . . . "On inspecting the throat, the uvula was found to be turned obliquely to one side, so that its point was directed towards the right amygdala; yet, when he inspired, the velum was elevated on both its sides. The eyelids were observed to be closed, as before, although their margins did not quite meet; and after elevating the upper eyelid, it descended again when he was asked to wink. But it was noticed that the eyelid did not descend with any degree of force; on the contrary, it presented a looseness and flaccidity which proved that the orbicularis was passive. On narrowly watching this motion, it was seen to depend, in a principal manner, upon the revolving of the globe of the eye, which takes place when we wink: for it was only after the cornea had been elevated, so as to be hid behind the raised eyelid, that the eyelid dropped over the white part of the eye." . . . "27th. Hitherto, owing to the general stupor with which he has been affected, it has been difficult to ascertain to what degree his hearing was injured on the right side. At one time it was our impression that the sense was entirely lost; but he now hears apparently quite well on this side, and only complains of a crackling noise in the ear.

"October 6th. When asleep, his eyelids were noticed to be perfectly closed; and he afterwards mentioned that it was without his consciousness that they were shut. A marked difference, however, is observed in their position when he is awake. A short time ago, when he lay comatose, they were always shut; and even after he recovered, the eyelids closed when he made the attempt to wink; but now they remain wide apart, and the upper one no longer descends, even if he winks with all his force. Indeed, it appears raised to a higher level than before."

Mr. Shaw enters into a somewhat minute consideration of the rationale of all the symptoms in this case, of the state of the eyelids and eye, the uvula, &c. We must confine our extracts to that portion of the paper which relates to the main affection of the nerve, and the ingenious explanation of the cause of the accident advanced by Mr. Shaw.

"The circumstance that gives the principal interest to this case, is the occurrence of partial paralysis of the face, in connexion with concussion of the brain. Previous to our improved knowledge of the functions of the nerves, such a combination could not have been explained in any satisfactory manner. No reason could have been assigned for certain muscles of the face alone being paralyzed, while others immediately adjoining those that were affected retained their power; nor conld it have been understood how any of the muscles of the face should have become paralyzed without the integuments being deprived of their sensibility. The only way of accounting for this kind of paralysis would have been by supposing that the injury to the brain, occasioned by the fall, had given rise to it. Yet such an explanation would only have increased the obscurity that already characterizes the symptoms resulting from blows on the head; for no lesion of the brain hitherto described has ever been found to produce a loss of power so limited in its nature as that presented in the foregoing case. When it is known, however, that each nerve of the brain is endowed with its appropriate function, it becomes an easy task to distinguish between the effects of a local injury to one of them, in its course from the brain, and the symptoms denoting a general disturbance of this important organ itself. Here, for example, no doubt can be entertained, that independently of the violence to the brain giving rise to the usual symptoms of concussion, the portio dura of the seventh pair has received an injury in some part of its course; and that this has occasioned the partial paralysis of the face. The portio dura, it has been ascertained, alone controls the particular muscles that have been paralyzed; it is known, also, that in its passage through the bones of the skull, it pursues a different route from the rest of the nerves; it can easily be understood, therefore, that it alone may have been injured, while the others escaped. Such an explanation will account for the sensibility of the skin, and also for the power over the muscles of the jaws remaining unimpaired, since neither of these properties depends upon the portio dura, but they are both conferred by the fifth pair, which takes a distinct course through the bones from that nerve.

"This case is a correct parallel to others that I have witnessed. Indeed, it may be stated that partial paralysis of the face, of the same kind as was exhibited in this patient, is not an uncommon effect of injuries of the head. It is, at all events, remarkable that from such accidents the portio dura is more liable to be injured than any of the other cerebral nerves. There is evidence of this in the recorded cases of affections of the different nerves of the head. If we refer to the papers on Partial Paralysis,* by Mr. John Shaw (containing the first illustrations submitted to the profession, of the practical benefits to be derived from the discoveries in the nervous system,) or to the collection of cases in Sir Charles Bell's work on the nerves, where a most extensive series of local nervous affections from different causes is given, it will be found that a considerable number of these resulted from blows received on the head. Ten such cases, at least, may be counted. Now in all of these the portio dura was injured; and in one alone, characterized by Mr. Shaw as uncommon in its occurrence, the fifth pair was partially involved in the injury, along with this nerve. Hence it is interesting to enquire, what is remarkable in the anatomy of the portio dura that should render it so peculiarly subject to have its function destroyed in these cases of blows upon the head. The explanation, as it appears to me, is to be sought for by attending to the course which this nerve takes through the bones of the cranium, and by bearing in mind, at the same time, the principle of *contre-coup*.

"It is by a circuitous route, through a canal of some length and complexity, that the portio dura pierces the temporal bone. Now, it is at this very part of the skull that we learn, both by experience and by studying the forms of the bones on the principle referred to, that the vibrations caused by a severe blow on the head are most powerfully felt: it is here that fissures produced by injuries received at distant parts of the skull are most frequently found. We cannot be surprised, therefore, when the portio dura (which, notwithstanding its name, is extremely delicate in its texture) has to pass through the temporal bone, enclosed in a narrow canal, with boundaries of such great density, that it should be peculiarly liable to have its function destroyed by the vibrations of the skull. Blows may be inflicted on the head, of every different degree of violence short of producing actual fracture of the bone, which will, at the same time, cause a considerable spurring out of the bone at the temple, and consequently a shock extending through the canal that contains the portio dura; and no one can venture to say what slight degree of concussion communicated to the nerve thus enveloped may not produce the immediate loss of its power; or by occasioning the effusion of blood, or of serum, or of lymph, around it, give rise to the subsequent destruction of its function. Just as hæmorrhage from the ear, which was one of the symptoms in this case, may either indicate a trifling laceration in the tympanum, or a fracture extending through the base; so may paralysis of the muscles of the features indicate either a slight or a formidable injury to the temporal bone. But let this opinion be correct or not, I conceive that I am borne out in saying, that the frequency with which paralysis of the face, hæmorrhage from the ear, and, I may add, effusion of blood at the temple from rupture of the meningeal artery, attend violent blows on the head, is to be accounted for by the tendency which the vibrations have, on the principle of *counter-fissure*, to be concentrated towards the temples.

"Without hazarding an opinion as to the exact nature of the injury received by the nerve, in this particular instance, I may be allowed to make a few observations on the subject. When we consider the kind of accident that befell the

* Quarterly Journal of Science, 1821; Medico-Chirurgical Transactions, 1822.

patient, the state of insensibility in which he lay for more than a week, and that the hæmorrhage from the ear lasted even for three days, we cannot help concluding that there must have been a fracture extending across the temporal bone, lacerating the portio dura in its canal. A fissure through part of this bone might have existed, even although the symptoms were only those of concussion. Nor will the recovery of the patient be offered as an argument against this opinion. I have, in the museum of the school, two interesting specimens of sculls, exhibiting fissures (one complicated with fracture) extending into the temporal bones in both instances; the fissures approach closely to the canal for containing the portio dura; yet it is obvious, from the signs of reparation, that the individuals from whom they were taken survived the accidents. But if there had been a fracture, in Kearsley, we should have expected to find, along with the hæmorrhage from the ear, some puffiness or discoloration of the integument at the temple. It may also be argued, that had the injury to the bone been of such a severe nature, the paralysis would have immediately succeeded the accident, instead of appearing, for the first time, on the fourth day. Besides, the manifest improvement in the condition of his face. shortly after the first accession of the paralysis, is inconsistent with the idea of the nerve having been torn through. It is to be presumed, likewise, that if the bone had been fractured to any extent, the portio mollis could scarcely have escaped; yet when the patient left the hospital, his hearing was scarcely, if at all, impaired.

"It will, perhaps, therefore, be more consistent with the facts observed in the case of Kearsley, if we consider that instead of there being a fissure extending completely through the bone, there was only, in consequence of the shock communicated by the fall, a partial breaking up of the thin partition in the interior of the temporal bone that divides the portio dura from the cavity of the tympanum. Such a fracture would be attended with laceration of the lining membrane of the tympanum, and might have given rise to the hæmorrhage from the ear, without being sufficient of itself to disturb the functions of the portio dura, so long, especially, as the blood continued to escape from the ear. When, however, the blood ceased to flow, it is probable that it accumulated in the tympanum, coagulated and pressed upon the nerve, thereby producing the paralysis. This corresponds with the manner in which the paralysis of the muscles took place; for it did not commence till the hæmorrhage from the ear ceased; and it corresponds also with the partial recovery of the patient, for it is not unlikely that the effused blood would be gradually absorbed in part." *Med. Gazette.* 1837.

---

*On an Undescribed Displacement of the Bones of the Fore-arm in Children.*
By John Gardner, Esq., Surgeon.

This is a case which most surgeons of experienee must have noticed. We call the attention of our younger readers to it; it is well described by Mr. Gardner.

"There is an accident of very frequent occurrence happening to children, from the time when they are just beginning to walk to the age of from three to four years. A parent or servant is leading a child, or it is supporting itself by its hand —a sudden slip occurs—a slight crack is heard—the child screams—and upon examination is found unable to use its hand; the arm hangs powerless by its side, or is supported by the other hand, and every attempt to move it is attended with considerable pain. A surgeon is summoned, and on the first aspect supposes that either the clavicle is fractured or the shoulder-joint dislocated. But when, on a careful examination, this is found not to be the case, and the non-existence of either dislocation or fracture is satisfactorily ascertained, he believes it to be a mere bruise, places the arm in a sling, and keeps it bathed with cold lotions. After some time, whilst dressing or undressing the child, or on some sudden movement, another fall, or pull upon the arm, a slight crack is again heard, and to the great surprise of the parent, the hand is forthwith used, and is found to be quite well.

"The displacement consists in the tubercle of the radius, to which the tendon of the biceps flexor cubiti is attached, slipping over the edge of the ulna, and being retained there. I have never seen this displacement in adults; probably the laxity of the ligaments permits it only in children, and most frequently in very young children. When a child is presented to me under these circumstances, after carefully ascertaining that there is no fracture either of the clavicle or bones of the arm, and no other dislocation, and the existence of this displacement being evident, I grasp the upper arm firmly in one hand, and with the other bring the fore-arm tightly supine, and suddenly bending the fore-arm upon the upper, the bones slip into their proper places: a slight crack is heard, and the child is well, and can at once use its hand. *Med. Gazette. September* 9, 1837.

---

### *On the Objections made to the Performance of Extraction in Cataract.*
By P. G. KENNEDY, M.D.

THIS communication is devoted to the consideration of the validity of Dr. Robertson's objections to contraction, of which we gave an account in our last volume, p. 257. It is a well written and well-reasoned paper, and it is due to Dr. Kennedy that we should notice it as well as Dr. Robertson's. The following extract from the conclusion of Dr. K.'s paper, gives a summary view of his opinions, and shews wherein he differs from Dr. Robertson.

"1st. Abstractedly considered, no particular operation for cataract is superior to another, because the forms which the disease assumes are so essentially different, and its complications with other ophthalmic and constitutional affections so numerous, that it is impossible to treat successfully all cases by any one particular plan.

"2d. The surgeon must therefore be able to apply each operation to those cases to which it may be suited; and the circumstance of extraction requiring the greatest degree of skill and dexterity can form neither a valid objection to its performance, nor an excuse for its non-performance, where the case may demand it.

"3d. When the case is one which leaves a choice to the surgeon, whether he shall perform reclination or extraction, the latter is to be preferred, because no objection to its performance is valid; and it is admitted by those to whom I am opposed, that, but for their supposed objections, it would be preferable.

"4th. It is absolutely necessary for the success of the operation in question, that the patient's constitution be sound, his general health good, and the eye, with the exception of the simple structural and functional alterations attendant on cataract, perfectly healthy." *Edinburgh Journal. October*, 1837.

---

### *On the division of the Tendo-Achillis in cases of Club-Foot.*
By JOHN WHIPPLE, Esq., Surgeon, Plymouth.

THIS is a very valuable practical paper, containing the details of no fewer than nine cases, all operated on by Mr. Whipple, and all with perfect success; if we except one in which the cure has been for the present defeated, owing to the occurrence of sloughing on the foot from improper management by the attendants after the operation. The patients were aged 9, 8, 28, 8, $2\frac{1}{2}$, $7\frac{1}{2}$, 14, 7, $1\frac{1}{6}$ years, respectively; and the comparison of the details justifies the deduction drawn by Mr. W. that infancy is the time most favorable for the operation. This surgeon's experience has so convinced him of the curability of this deformity by the operation when properly conducted, that he asserts "that every case of talapes verus, if not arising from cerebral or spinal irritation, can be cured by steady attention and perseverance on the part of the surgeon; and that to him alone, and not to the operation itself, is all blame due if he fails of success."

The following extract contains Mr. Whipple's account of his manner of operating, and his reasons for the particular plan adopted by him.

"The foot being extended as much as possible, the integument posterior to the tendon is pinched up about two inches above the os calcis, in order to separate it from the latter, when a narrow-bladed knife, with a rounded cutting extremity, is passed from within obliquely downwards and outwards; between the integument and tendon; and as soon as the point of the knife is felt under the integument, and on the outer side of it, considerable flexion of the foot is made by an assistant, the point of the knife being at the same time depressed, so as to bring it in contact with the tense tendon, when, by firmly depressing and withdrawing the instrument, the object is instantly effected. This is made evident by the sudden jerk with which the heel is brought down, in some instances two or three inches, as in cases of talapes equinus. The knife should be passed from the inside outwards, for this reason: should you depress the point more than is necessary to divide the tendon, there would be no risk of wounding the posterior tibial artery, which would be the case were you to introduce your knife from without inwards; and it is essential to depress with some force, or you leave undivided some fibres of the tendon most remote from your puncture, and have to introduce your knife again (not a little embarrassed at your own bungling) for the purpose of dividing them. However, although the point of your knife be dipped some distance anterior to the edge of the tendon externally, in order to secure its division, this will not be necessary internally, as, the moment you feel your object effected, you discontinue the pressure on the knife, and withdraw it carefully, so as not to enlarge the integumental opening.

"This, I think, is by far the best mode of operating, as by this means you pass your knife across a relaxed tendon, which, when rendered tense, is brought up to meet the edge of the instrument, and therefore more readily divided than when you pass your knife between it and the deeply-seated muscles. Another objection to the latter plan with me is, that the tendon is in such close contact with the integument, that you run a great risk of dividing, or partially dividing, the latter, which, from the years of contraction to which it has been subjected, is rendered exceedingly tense when the foot is flexed. In upwards of thirty cases which I have examined, I have found no exception to this. Again, where the toes are the points of support, the tendon will be found nearly embraced by the integument, as in the corresponding tendon in the horse, though certainly not to such an extent. I must not leave this part of the subject without a remark relative to the division of other tendons apparently implicated, without the division of which it might be imagined that little would be gained; and, indeed, such was my own impression after the operation in the second case I have recorded. I had promised that one tendon only should be divided; but I confess that I left my patient with regret at having so given my word, and determined to gain the consent of the parents to the division of the others, if the muscles did not elongate by steady and constant extension, as I at first conceived they would, looking upon them as secondarily affected, their contractility being favoured by the rolling inwards of the foot. A few days, however, served to remove all doubt from my mind, as they were evidently relaxing. I abandoned then the idea of their division being necessary, and as yet I have had no occasion to regret it. I am free, however, to acknowledge, that it might be the means of a more speedy alteration of the shape of the foot; yet the chances of inflammation, together with the weakness which a want of union would necessarily induce, are sufficient reasons for its division not being attempted. No doubt can exist of its impropriety in cases of talapes verus, as will be illustrated hereafter.

"My reasons for dividing the tendon obliquely are as follows: First, by so doing you have a larger surface for nature to carry on her operations; secondly, you have the obliquely divided tendon in nearer approximation, and thereby secure a firmer ligamentous band than in the transverse division; and thirdly, the application of the instrument does not separate the lips of the wound—a desirable point, as the sooner it heals, so as to prevent the escape of lymph, the better. The puncture is dressed with adhesive plaster, and the instruments applied at once, as, where this has been deferred, the act of stretching the inflamed part has caused considerably more pain than the operation and early application combined. Much care and attention are required for the first three weeks or month, in order to keep the heel well down. Every thing depends on the heel and instep straps, and neither the fears and doubts of the surgeon, nor the ill-timed meddling of the parents, must interfere with the application of these straps; for, however aggravated the case may be, the removal of the deformity by proper treatment is certain. I know of no instance where patience is more necessary to the surgeon than in treating these cases; every thing is to be gained by it; for, by strapping too tightly and screwing too firmly, vesications are produced, which compel you to remove every thing for their cure, and you lose more in twenty-four hours than you have gained in a week. Therefore, all you can do is to secure the heel firmly to the iron-sole, and to screw the plate so that it may merely rest on the cuboid and tarsal bones; then, from day to day, to draw in the strap a little tighter, so as to bring the end of the splint to the knee: a little pain and inconvenience are of course attendant upon this proceeding, but provided it does not produce vesication, this cannot be of any consideration when put in competition with the importance of the result."

*Med. Gazette. September* 2, 1837.

---

*On the Use of Nitrate of Silver in some Cutaneous Diseases.*
By HENRY T. CHAPMAN, Esq. Surgeon.

OUR readers are well aware of the use made of this remedy in erysipelas, &c., first recommended by Mr. Higginbottom. The present valuable paper contains practical remarks on its use in such cases and in others not contemplated by the original proposer. The following extracts contain the more important results obtained by Mr. Chapman.

"Of erythema nosodum two cases were treated: in one of them the redness and induration disappeared entirely after a single application of the caustic; in the other it was necessary to repeat it once more. My next experiment was made on squamous affections of the skin; lepra, namely, and several varieties of psoriasis. I possess the details of more than a dozen cases successfully treated by it, and have since employed it with equal success in a great number of instances, of which I have neglected to take notes."

"Besides the squamous disorders above mentioned, I have made trial of the nitrate of silver upon other cutaneous diseases. In porrigo, a strong solution of it is recommended by some practitioners almost as a specific. It was, I believe, a favorite remedy with Mr. Wilkinson, who acquired some celebrity for his treatment of this troublesome malady. I have tried it repeatedly both in solution and in the solid state, but in neither form was any permanent advantage gained by it. In sycosis menti and eczema, in both of which disorders, as well as in herpes zoster, its efficacy has been rated highly, I have been equally disappointed. In short, as far as my experience of its beneficial effects in diseases of the skin extend, it exerts a decided influence over those of a squamous character alone."

The following is the mode of applying the caustic as originally proposed by Mr. Higginbottom:

"After cleansing the skin with soap and water, and drying it, the surface to be submitted to its operation is again moistened, and a solid stick of the lunar caustic is rubbed lightly over it once or twice, according to the delicacy of the skin, and allowed to dry. No dressing is required, and the part must be kept cool. The slight vesication produced after the first twenty-four hours soon subsides, and about the fifth day the black pellicle loosens and peels off."

Our younger readers must not adopt this practice empirically or regard it as specific. Mr. Chapman is far from doing so, as the following sensible remarks show:

"In squamous disorders of the skin, besides the separation of the loaded cuticle, the nitrate appears to correct the diseased action of the capillaries, on which the accumulation depends, and restore a healthy tone to the vessels. This result, however, can never be expected, while the original source of the disease still exists in the system; and before the local remedy can produce any permanent benefit, constitutional measures must be steadily enforced. Some may exclaim that the cure, in that case, is unjustly ascribed to the nitrate; but is it not highly probable that the diseased product still continues to be formed habitually, long after the state of health giving rise to such an excretion has been completely rectified? In these affections, therefore, the nitrate of silver is only to be looked upon as an auxiliary to other remedial means, which must always occupy the foremost place."

*Med. Gazette. October* 14, 1837.

### *Cure of congenital Club-Foot by division of the Tendo-Achilis.* By Thomas Inglis, m.d., Glasgow.

The interesting case here related is that of the narrator, and the operation was performed by Dr. Little, on the 11th of August last. At the date of the present communication (3d October, 1837,) Dr. Inglis says, "I have been walking about for the last four weeks. I may now say that I am not only free from deformity, but enjoy an ease, freedom, and power in locomotion, such as it was never my lot to enjoy at any previous period of my existence."

*Lancet. October* 21, 1837.

